# Linking Network Activity to Synaptic Plasticity during Sleep: Hypotheses and Recent Data

**DOI:** 10.3389/fncir.2017.00061

**Published:** 2017-09-06

**Authors:** Carlos Puentes-Mestril, Sara J. Aton

**Affiliations:** Neuroscience Graduate Program, Department of Molecular, Cellular, and Developmental Biology, University of Michigan Ann Arbor, MI, United States

**Keywords:** homeostatic plasticity, replay, synaptic homeostasis hypothesis, oscillations, REM sleep, NREM sleep

## Abstract

Research findings over the past two decades have supported a link between sleep states and synaptic plasticity. Numerous mechanistic hypotheses have been put forth to explain this relationship. For example, multiple studies have shown structural alterations to synapses (including changes in synaptic volume, spine density, and receptor composition) indicative of synaptic weakening after a period of sleep. Direct measures of neuronal activity and synaptic strength support the idea that a period of sleep can reduce synaptic strength. This has led to the synaptic homeostasis hypothesis (SHY), which asserts that during slow wave sleep, synapses are downscaled throughout the brain to counteract net strengthening of network synapses during waking experience (e.g., during learning). However, neither the cellular mechanisms mediating these synaptic changes, nor the sleep-dependent activity changes driving those cellular events are well-defined. Here we discuss potential cellular and network dynamic mechanisms which could underlie reductions in synaptic strength during sleep. We also discuss recent findings demonstrating circuit-specific synaptic strengthening (rather than weakening) during sleep. Based on these data, we explore the hypothetical role of sleep-associated network activity patterns in driving synaptic strengthening. We propose an alternative to SHY—namely that depending on experience during prior wake, a variety of plasticity mechanisms may operate in the brain during sleep. We conclude that either synaptic strengthening or synaptic weakening can occur across sleep, depending on changes to specific neural circuits (such as gene expression and protein translation) induced by experiences in wake. Clarifying the mechanisms underlying these different forms of sleep-dependent plasticity will significantly advance our understanding of how sleep benefits various cognitive functions.

## The role of sleep in cognition: an unsolved mystery

Nearly a hundred years of behavioral research indicates a role for sleep in human cognition. Short-term (i.e., hours-long) sleep deprivation (SD) is known to lead to deficits in performance on memory, sustained attention, and perceptual tasks in human subjects (Killgore, 2010; Krause et al., 2017). Longer-term (i.e., days-long) SD can cause profound cognitive disruption (Meyhofer et al., 2017). In animal models, various neurocognitive performance deficits have been described following SD (Aton, 2013; Havekes and Abel, 2017). This has led to the hypothesis that at least some forms of synaptic plasticity associated with these cognitive processes occur preferentially during sleep. Recent evidence from both animal models and human subjects has supported this idea. For example, both anatomical (Yang, G. et al., 2014; Havekes et al., 2016) and functional (Aton et al., 2009a, 2013; Durkin and Aton, 2016) remodeling of cortical circuitry after a novel experience occurs selectively during sleep, and is blocked by SD.

Thus for neuroscientists, a critical question is: how does sleep promote nervous system plasticity? Addressing this question has proven difficult. First, as we will discuss here, sleep may promote different forms of plasticity under different environmental circumstances. Thus, the effects of sleep (and SD) on the brain may vary with the cognitive demands of an animal's present circumstances. Second, the underlying mechanisms driving sleep-dependent plasticity have been elusive. In part, this is because sleep and wake states alter so many aspects of brain physiology simultaneously—neurotransmission, neuromodulation, transcription, translation, neuronal and network activity, interstitial space and ion concentration, etc. (Aton, 2013).

## Part I: the synaptic homeostasis hypothesis

### What is the synaptic homeostasis hypothesis?

Few hypothetical mechanisms have been proposed with an aim toward explaining the many neurocognitive effects of sleep and SD. One notable exception is the sleep and synaptic homeostasis hypothesis (SHY) (Tononi and Cirelli, 2003). SHY has been proposed as an all-encompassing mechanism to explain why cognitive deficits result from sleep loss. SHY proposes that during wake net synaptic strength increases throughout the brain as a function of experience-dependent plasticity; over time this leads to alterations in energy utilization, reductions in space for further plasticity, and disrupted information processing by neurons. SHY further posits that during sleep, synapses throughout the brain are globally reduced in strength (i.e., “downscaled”) to offset wake-associated synaptic potentiation. This process is hypothesized to conserve energy, improve the signal-to-noise ratio in neural circuits, avoid saturation of synaptic strength, and prevent pathological levels of excitation in neurons (e.g., epilepsy); it has thus been touted as “the price of plasticity” (Tononi and Cirelli, 2014) by proponents of SHY.

Here we discuss SHY in the context of what is currently known regarding the physiology of the brain during sleep. We will review recent data which either support a SHY-based mechanism for sleep-dependent plasticity, or provide a potential counterpoint to SHY. We also discuss other hypothetical sleep-specific mechanisms which could support brain plasticity.

### What is the evidence for sleep-dependent reductions in synaptic strength?

**Figure 1:**
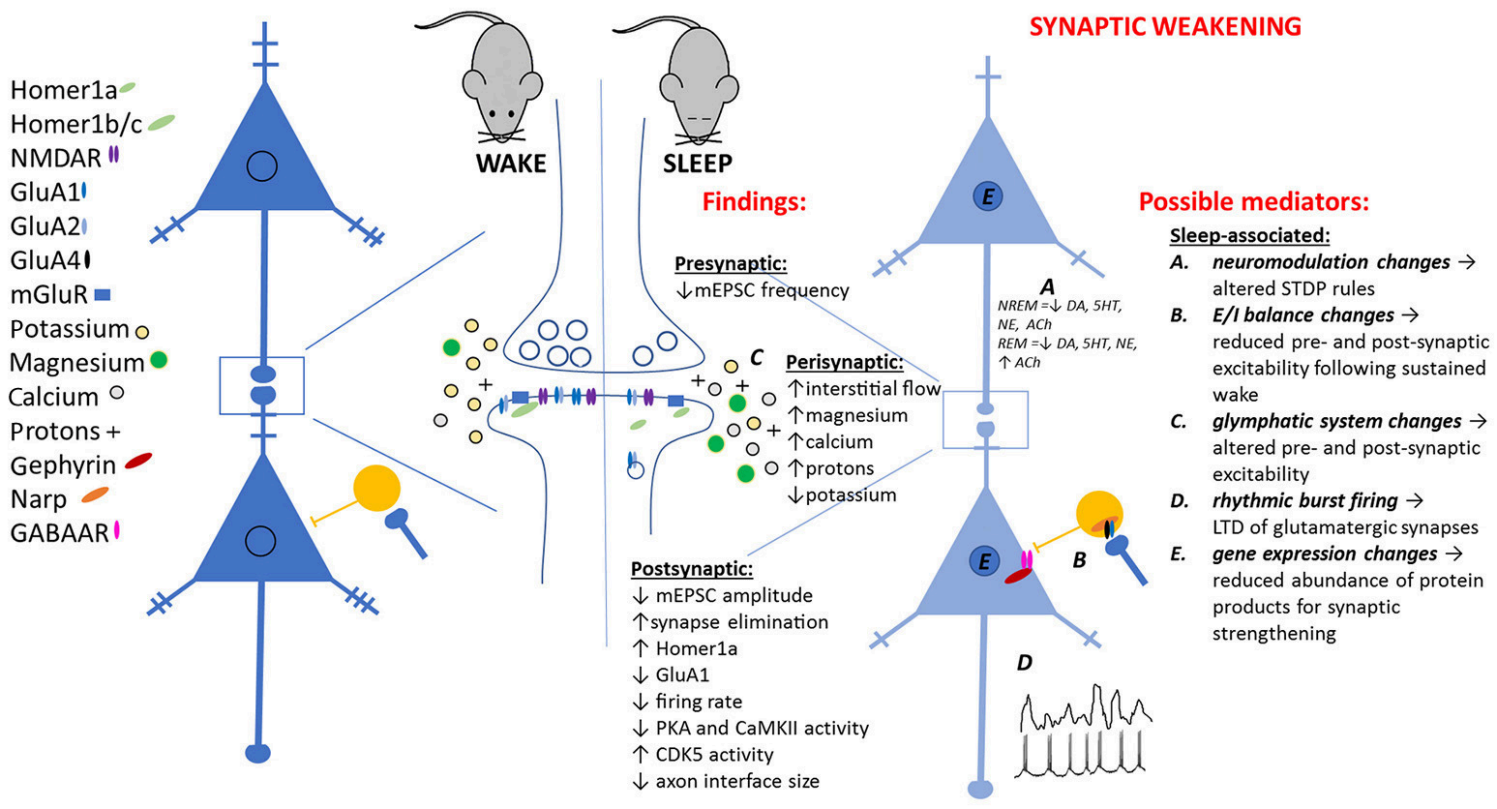
Observed pre- and post-synaptic changes attributed to sleep-dependent synaptic weakening, and potential sleep-dependent mechanisms.

Since SHY was first proposed, data to support the hypothesis have come from biochemical (Vyazovskiy et al., 2008), electrophysiological (Vyazovskiy et al., 2009), and anatomical (de Vivo et al., 2017) studies of the effects of brief SD or *ad lib* sleep. These data are outlined in Table 1 and Figure 1, and are described in detail below.

**Table 1:** Summary of evidence in support of sleep-associated synaptic weakening, and sleep-associated synaptic strengthening.

**Manipulation**	**Key findings**	**Species**	**Age**	**Brain area**	**References**
**EVIDENCE FOR SYNAPTIC WEAKENING DURING SLEEP**					
**Biochemistry**					
5 h sleep vs. SD^a^	Induction of *arc, cfos*, and *creb* during SD	Mouse	2–4 months	Hippocampus	Vecsey et al., 2012
3–12 h sleep vs. SD^a^	*narp, cfos*, and *bdnf* induced during SD in cortex	Mouse	10 weeks	Cortex (somatosensory and motor) and hypothalamus	Mackiewicz et al., 2007
Sleep (ZT8), 8 h SD^b^ (ending at ZT 8), wake (ZT20)	During wake and SD, *bdnf* and *narp* induced in cortex and cerebellum; *homer1a, cfos*, and *arc* induced in cortex	Rat	Unknown	Cortex (unknown areas) and cerebellum	Cirelli et al., 2004
Sleep (ZT6), 6 h SD^c^ (ending at ZT 6), wake (ZT18)	During wake and SD, ~20% increase in GluA1, pCaMKIIa, and pSer845-GluA in synaptoneurosomes from both areas	Rat	12–14 weeks	Cortex (unknown areas) and hippocampus (synaptoneurosomes)	Vyazovskiy et al., 2008
Sleep (ZT4), 4 h SD^d^ (ending at ZT 4), wake (ZT16)	~20% increase in post-synaptic GluA1, pSer845-GluA, and PKA at ZT16 relative to ZT4, no changes with SD	Mouse	8–10 weeks	Forebrain (synaptosomes)	Diering et al., 2017
**Anatomy**					
2 h sleep vs. SD^e^	Spine/filopodia formation equal between sleep and SD, elimination increased ~10% across sleep relative to SD	Mouse	3 weeks	Somatosensory cortex, layer 5 pyramidal neurons	Yang and Gan, 2012
Sleep (ZT6), 6 h SD (ending at ZT 6), wake (ZT18)^f^	During wake and SD, axon spine interface size increased ~10–15% (only affected smaller spines; largest unaffected)	Mouse	4 weeks	Primary motor and somatosensory cortex	de Vivo et al., 2017
**Electrophysiology**					
ZT1 vs. ZT5-6, 4 h SD (ending at ZT4)^g^	Decreased firing rates in fast-spiking interneurons at ZT5-6 vs. ZT0, increased multiunit firing after SD	Rat	13–16 weeks	Barrel cortex and frontal cortex	Vyazovskiy et al., 2009
4 h sleep vs. SD^a^	increased mEPSC amplitudes and frequencies after SD	Mouse/Rat	3–4 weeks/4–8 weeks	frontal cortex	Liu et al., 2010
Spontaneous sleep and wake bouts	Firing rates increase across wake and decrease across sleep; ratio of interneuron-to-pyramidal neuron firing higher during wake than sleep	Rat	Unknown (adult)	Hippocampal area CA1	Miyawaki and Diba, 2016
**EVIDENCE FOR SYNAPTIC STRENGTHENING DURING SLEEP**					
**Biochemistry**					
1 h sleep vs. SD^h^ following monocular visual experience	Increased synaptic BDNF protein levels during sleep (but not SD); decreased Arc protein levels after SD	Cat	Post-natal day P28-40	Primary visual cortex (homogenate and synaptoneurosomes)	Seibt et al., 2012
1 or 2 h sleep vs. SD^h^ following monocular visual experience	5–10 fold increase in pCaMKIIa, pERK, and pSer831-GluA1 during sleep (but not SD)	Cat	Post-natal day P28-40	Primary visual cortex (homogenate)	Aton et al., 2009a
1, 3, or 6 h of *ad lib* sleep following two-way active avoidance or sham training	post-training increases in pCREB, BDNF and Arc protein proportional to post-training increases in REM PGO wave density	Rat	Unknown (adult)	Hippocampus, amygdala, frontal and occipital cortex (homogenate)	Ulloor and Datta, 2005
**Anatomy**					
5 h sleep vs. SD^h^	~20% decrease in spine density after SD	Mouse	2–3 months	Hippocampal area CA1 pyramidal neurons	Havekes et al., 2016
~7 h sleep vs. SD^a^ following motor learning	~50% decrease in spine formation across period of SD relative to sleep	Mouse	Unknown (adult)	M1 layer 5 pyramidal neurons	Yang, G. et al., 2014
**Electrophysiology**					
Spontaneous sleep and wake bouts	Increased amplitude evoked field potential responses following NREM sleep	Cat	Unknown (adult)	Somatosensory cortex	Chauvette et al., 2012
3 or 5 h sleep vs. SD^i^	Disruption of PKA-dependent forms of LTP after SD	Mouse	2–4 months	Hippocampal area CA1	Vecsey et al., 2009; Prince et al., 2014
6 h sleep vs. SD^i^ following novel visual experience	Selective firing rate responses increased after sleep (but not SD); neuronal firing rates increase across bouts of NREM and REM (but not wake)	Mouse	1–4 months	Primary visual cortex	Durkin and Aton, 2016

a *SD via tactile stimulation*.

b *SD via air puffs, exposure to novel objects*.

c *SD via exposure to novel objects*.

d *SD via cage change*.

e *SD via exposure to novel objects and gentle touch*.

f *During both SD and wake phase (not sleep phase) mice were given access to a running wheel and exposed to novel objects*.

g *SD via exposure to novel objects and acoustic stimuli*.

h *SD via novel objects, acoustic stimuli, tactile stimulation, and floor rotation*.

i *SD via cage tapping, shaking, and nest disturbance*.

#### Gene expression

Early studies that aimed to clarify the functions of sleep in the brain focused on gene expression changes following brief (i.e., hours-long) periods of sleep or SD. These studies assessed changes in mRNA levels in different parts of the brain—hypothalamus (Terao et al., 2003), neocortex (Cirelli et al., 2004; Mackiewicz et al., 2007), cerebellum (Cirelli et al., 2004), and hippocampus (Vecsey et al., 2012)—using microarray analysis. Across these studies, a consistent finding is that the expression of genes involved in RNA, protein, and lipid biosynthetic pathways, the unfolded protein response (UPR), and synaptic plasticity change as a function of sleep and wake. More specifically, sleep is associated with increased expression of genes associated with protein and lipid synthesis, while SD is associated with increased expression of genes involved in mRNA transcription, cellular stress and the UPR. In support of SHY, in many of these studies, sleep decreases and wake increases expression of a subset of genes thought to be involved in activity-mediated synaptic plasticity—including *arc, cfos, bdnf*, *narp*, and *homer1a*. More recently, the Allen Brain Institute has made microarray and *in situ* hybridization data available from numerous regions in sleeping and sleep-deprived animals, revealing a more complex picture of gene regulation (i.e., across the entire brain) during different behavioral states (Wang et al., 2010). These gene expression data have been used as support for the idea that activity-mediated synaptic plasticity is widespread in the brain during wake, and generally reduced during sleep.

#### Synaptic protein expression

A critical unresolved question is whether the levels of protein translated from sleep- and wake-regulated mRNAs are similarly altered by state. Changes in protein levels appear to track transcript level changes in some of the cases where it has been carefully investigated (Cirelli et al., 1996; Simor et al., 2017). However, state-dependent changes in protein synthesis (Ramm and Smith, 1990) may compensate for some changes in gene expression during SD. For example, in the hippocampus, levels of *arc* and *hspa5/BiP* mRNA increase across a brief period of SD; however, due to alterations in translation rates during wake, levels of Arc and Hspa5/BiP protein abundance remain unchanged (Tudor et al., 2016).

Despite these caveats, recent studies have found evidence in support of SHY based on synaptic protein expression. In rats, for example, expression of GluA1 and active (phosphorylated) CaMKII is increased by roughly 20–40% in cortical and hippocampal synaptoneurosomes following a 6-h period of SD, relative to a similar period of sleep (Vyazovskiy et al., 2008). A more recent study (Diering et al., 2017) reported a similar ~20% decrease in GluA1 and GluA2 content in mouse forebrain synaptosomes during the circadian sleep phase relative to the wake phase. Critically, however, the authors were unable to replicate the previously-reported effects of SD on these targets (i.e., synaptic GluA1 and GluA2 levels were identical with sleep, SD, and SD + recovery sleep). Nonetheless, the authors conclude based on these data that a global downscaling mechanism acts on synapses during sleep (Diering et al., 2017).

#### Synaptic morphology

Effects of sleep have also been seen at the level of dendritic structure in the developing brain. Yang and Gan (2012) recently used *in vivo* imaging of layer 5 pyramidal neurons' dendrites in the somatosensory cortex of juvenile mice, to investigate the effects of brief (i.e., 2-h) periods of sleep and SD on spine turnover. The authors found that across 2 h of SD, total dendritic spine/filopodia density increased by ~5%, while across 2 h of *ad lib* sleep, it decreased by ~5%. The difference was apparently due to increased elimination rates for existing spines and filopodia during sleep (there was no difference in the rate of new spine formation between sleep and wake). More recently, serial scanning electron microscopy (SEM) was used to reconstruct and measure dendritic spines (and apposed axon termini) in layer 2 of primary motor and somatosensory cortex of juvenile mice after periods of wake (spontaneous or enforced) vs. sleep (de Vivo et al., 2017). By quantifying the surface area of thousands of individual contacts between axon terminals and spines, the authors concluded that sleep leads to a small (~10–15%) but significant decrease in synaptic contact area. This effect is heterogeneous, with the largest synaptic contacts unaffected by sleep vs. wake. More modest effects of sleep are seen on the size of dendritic spines themselves (e.g., spine volume), with only a small subset of spines quantified showing any effect of sleep vs. wake. While the function consequence of these changes remains unclear, proponents of SHY have pointed to these findings as the most direct evidence that synaptic strength is reduced during sleep.

#### Neuronal activity levels and excitatory/inhibitory balance

Numerous recent studies have used neuronal firing rates in freely behaving animals as surrogate measure for (or potential functional readout of) synaptic strength. While this measure is indirect, and can also be affected by changes in the intrinsic excitability, many laboratories have used it as a potential indicator of overall synaptic strength (Vyazovskiy et al., 2009; Aton et al., 2013, 2014; Hengen et al., 2013, 2016). For example, Vyazovskiy et al. tracked firing rates of rat barrel cortex neurons across periods of sleep and wake, and across the circadian day (Vyazovskiy et al., 2009). In this study, the authors found that neurons tended to fire at a lower rate at the end of the day (when “sleep pressure”—i.e., the propensity to fall asleep—is low) compared with the beginning of the day (when sleep pressure is high). Assuming that firing rate was directly proportional to (excitatory) synaptic strength, the authors concluded that these data indicated that greater synaptic strength is associated with greater sleep pressure, and that sleep reduces synaptic strength (in support of SHY). Importantly however, while these effects on firing rate were present both at the level of multiunit activity and in single neurons identified by the authors as fast-spiking interneurons, they were not statistically significant in putative pyramidal neurons. Nonetheless, this was the first demonstration of a functional change in neural circuits that could be related to the proposed mechanism in SHY.

If we assume (based on these findings) differential effects of sleep on firing in fast-spiking interneurons and pyramidal neurons, one possibility is that excitatory/inhibitory balance (i.e., the ratio of activity in glutamatergic and GABAergic neurons) is the major feature of cortical physiology that changes with sleep pressure. In support of this idea, a recent study of the hippocampal neurons' firing across states found the highest ratio of interneuron firing -to- pyramidal neuron firing during active wake (Miyawaki and Diba, 2016). Vanini et al. (2012) recently demonstrated that the rate of GABA release in the cortex increases steadily across periods of sustained wake. Another recent study found that while glutamate release in rat somatosensory and motor cortex also increases across brief periods of spontaneous wake, with SD, extracellular glutamate levels initially rise (over a period of 30 min- 2 h) and then fall (Dash et al., 2009). This supports the idea that sustained wake leads to a gradual decrease in excitatory/inhibitory balance associated with increasing inhibitory neurotransmission.

#### Synaptic physiology

Additional evidence suggests that synaptic function *per se* may be altered after sleep vs. wake. For example, Liu et al. recently measured the frequency and amplitude of mEPSCs in layer 2/3 pyramidal neurons of juvenile rat and mouse frontal cortex after periods of sleep and wake (Liu et al., 2010). While the authors concluded that a 4-h period of SD significantly increased both mEPSC amplitude and frequency, it is worth noting that values for both sleep and wake groups were highly variable. For example, depending on the set of experiments in the study, sleep deprived and sleeping groups' mEPSC frequencies were either quite distinct, or completely overlapped (Liu et al., 2010). Furthermore, while frequency changes (presumably reflecting effects on presynaptic release of glutamate) were relatively large, mEPSC amplitude changes (which would be affected by post-synaptic changes in receptor expression) were minimal across sleep vs. wake or SD. However, to date, this is the most direct evidence of functional synaptic weakening across a period of sleep.

#### Caveats regarding the evidence supporting SHY

The data outlined above has been put forth by proponents of SHY as evidence of sleep-dependent downscaling, which renormalizes synapses following changes in neural circuits (i.e., synaptic potentiation) caused by wake-associated learning. One major caveat is that many of the studies described above (and all of the studies describing anatomical changes) were carried out in adolescent animals (see Table 1). As is true for humans (Tang et al., 2014), the rate of synapse elimination in both adolescent rats (corresponding to post-natal weeks 5–9; Drzewiecki et al., 2016**)** and mice (corresponding to post-natal weeks 4–8; Zuo et al., 2005a,b; Bian et al., 2015) is maximal, and significantly higher than that seen in the adult brain. An unanswered question is whether sleep plays a special role in promoting developmentally-regulated synapse downscaling and elimination in adolescence, or whether sleep-dependent synaptic effects are present across the lifespan. Effects of sleep on synaptic structure and function in the adult brain are still far from clear.

A second caveat is that in many of the studies supporting SHY, comparisons between sleeping and awake animals are confounded by one of two factors. Either (1) samples come from animals spontaneously asleep or awake at different circadian times, or (2) SD animals used for comparison have been deprived of sleep through environmental enrichment (e.g., novel object or running wheel presentation) that was not provided to sleeping animals (see footnotes in Table 1).

A third major caveat is that while SHY proponents have used a global downscaling mechanism to explain neural network performance improvement using computational models (Hill et al., 2008), biological data have not supported the idea of global downscaling during sleep. For example, cortical and hippocampal neurons show non-uniform changes in firing rate across bouts of sleep (Miyawaki and Diba, 2016; Watson et al., 2016). Specifically, neurons with the highest baseline firing rates (including interneurons) show decreases in spontaneous activity across periods of NREM sleep, while neurons with lower baseline firing rates show either no change, or an increase, in spontaneous firing across a period of sleep. This indicates that functionally, not all neurons are equally affected by sleep, and it stands to reason that not all synapses are equally affected. Indeed, as described above the available anatomical evidence indicates that only a subset of synapses show a reduction in size across a period of sleep (de Vivo et al., 2017). Based on these new findings, the use of the term “synaptic downscaling” may itself be questionable, as sleep does not appear to have truly global effects with regard to reducing synaptic strength (i.e., “scaling” may not be present).

For this reason, more recent descriptions of SHY have proposed that sleep leads to a decrease in the strength *of only a subset* of synapses, while preserving the strength of others. This preservation would be highly desirable for processes involved in learning and long-term memory formation, where information encoded by neural circuits prior to sleep needs to be retained or reinforced. Given these findings, a critical question is why during sleep, some synapses (and possibly some neurons) are apparently unaffected, while others undergo an apparent reduction in strength.

Finally, in none of these studies were the observed changes linked with sleep-dependent cognitive function. The animals under study were housed in standard (i.e., non-enriched, and presumably non-challenging) conditions, and were not being trained on specific learning tasks. While sleep affects numerous aspects of cognition (including experience-dependent sensory plasticity and memory consolidation, described in detail below) that are affected by sleep, sleep's effects on these processes have not been linked to synaptic weakening. Thus, while converging data suggest that under steady-state conditions, *modest* weakening *of at least some* synapses can been observed in multiple brain areas across periods of sleep, the function of this for information processing in the brain (if any) is still unknown.

### What sleep-dependent mechanisms could mediate synaptic weakening?

A past major criticism of SHY is the lack of a specific, sleep-dependent, cellular mechanism mediating the observed biochemical and electrophysiological changes (Frank, 2013). Here we critically evaluate some hypothetical cellular and network mechanisms (see Figure 1) for these observations.

#### Neuromodulatory biasing of spike timing-dependent plasticity (STDP)

Recent computational modeling studies from the Tononi lab (Olcese et al., 2010; Nere et al., 2013) invoked a modified STDP rule to explain reductions in synaptic strength during sleep, and the effects of this process on memory. The STDP rule employed dictated that during learning in the wake state, synapses with temporally correlated pre- and post-synaptic firing would be strengthened, while synapses with non-correlated firing would either be unaffected, or would be weakened. In contrast, during sleep, synapses with temporally correlated pre- and post-synaptic firing would be unaffected (i.e., their strength would be preserved), while synapses with non-correlated firing would be weakened. In the earlier study, this was implemented computationally by simply inverting the sign of STDP normally seen the cortex (Feldman, 2000). As implemented in this scheme, the same spike timing would cause LTD instead of LTP, for the same pre-post-activity pairing, if it was present in sleep instead of wake. The authors argued that the presence or absence of neuromodulators (a function of brain state) would result in the same pattern of firing having differential effects on synapses in the two states. This model was meant to illustrate the benefits of sleep-dependent reductions in synaptic strength, rather than to clarify the cellular mechanisms in operation *in vivo*. However, it is necessary to point out that the proposed cellular mechanism is at odds with neurobiological data in two important ways.

First, sleep and wake are not monolithic with regard to neuromodulation, nor is the neuromodulation state of the cortex binary. Dopamine, serotonin, acetylcholine, and norepinephrine release rates are differentially regulated by state, and these effects vary according to where in the brain release is being measured (Marrosu et al., 1995; Portas et al., 1998; de Saint Hilaire et al., 2000; Lena et al., 2005). Second and more importantly, the effects of the state-regulated neuromodulators dopamine, acetylcholine, and norepinephrine on STDP do not support the notion that STDP rules “flip” between wake and NREM sleep. Each neuromodulator has distinct effects on the relationship between spike timing and synaptic strength changes (Pawlak et al., 2010), however, none of these effects fit with the assumptions of the model's modified STDP rule. For example, acetylcholine (with cortical release highest during wake, intermediate during REM and lowest during NREM) (Marrosu et al., 1995) can block timing-based LTP and promote timing-based LTD of glutamatergic synapses in cortical pyramidal neurons (Seol et al., 2007). In contrast, noradrenergic signaling (with cortical release highest during wake, intermediate during NREM and lowest during REM) promotes timing-based LTP in both cortical pyramidal neurons and interneurons. These effects are independent of the relative timing of action potentials and EPSPs—i.e., neuromodulator tone, but not the ordering of pre- and post-synaptic activity, determines the outcome of spike pairing. Taken together, available data suggest that higher norepinephrine and acetycholine levels during wake would lower the threshold for inducing *both* STDP-based LTP *and LTD*. It is therefore unlikely that changes in neuromodulation alone would bias plasticity in favor of LTD during sleep.

#### Homeostatic synaptic downscaling

Central to SHY is the concept of a globally-acting homeostatic mechanism which maintains synaptic strengths within a set physiological range. Homeostatic synaptic downscaling is a mechanism of plasticity that is thought to function in exactly this way, to counteract the network-level effects of excessive neuronal activity and synaptic excitation. Homeostatic downscaling differs from Hebbian synaptic weakening (e.g., LTD) with regard to both mechanism of induction and function. While LTD induction requires appropriately timed pre- and post-synaptic firing, and can lead to functional changes within minutes to hours, homeostatic downscaling appears to require increased neuronal firing and acts over a slower timescale of several hours to days. Homeostatic downscaling was first described *in vitro* by (Turrigiano et al., 1998), who described divisive shifts in neurons' mEPSC amplitude distributions in response to long-term increases in firing. Specifically, the authors found that 48 h of exposure to the GABAA receptor antagonist bicuculline led to a global reduction in neurons' mEPSC amplitude distribution (to ~66% of baseline) (Turrigiano et al., 1998). This study, along with numerous others since its publication, have led our current understanding of downscaling, wherein perturbations in either neuronal firing rate or neurotransmission leads to a global reduction of post-synaptic strength over several hours to days. Functional decreases in synaptic strength due to downscaling are accompanied by decreases in glutamatergic receptor (e.g., AMPAR) expression and spine volume (Turrigiano et al., 1998; Fernandes and Carvalho, 2016; Keck et al., 2017).

Only recently has sleep been implicated in regulating molecular pathways involved in homeostatic downscaling. Homeostatic reductions in AMPA receptor expression are mediated through multiple cellular pathways, and there is evidence that these pathways may be affected in parallel by sleep. Recent phosphoproteome profiling indicates that a kinase critical for downscaling, cyclin dependent kinase 5 (CDK5), is more active in the brain during the sleep phase of the rodent circadian cycle (Diering et al., 2017). CDK5 activity is increased in the nucleus of neurons in response to increased network activity (Liang et al., 2015), and is implicated in numerous cellular pathways that could promote synaptic downscaling. Within the nucleus CDK5 phosphorylates many targets including MeCP2. This phosphorylation event is critical for decreasing *gluA2* mRNA expression in response to an increase in neuronal activity. CDK5 also interacts with polo like kinase 2 (PLK2) to promote downscaling via downstream effects on the Rap GTPase pathway. This leads to regulation of Rap-mediated changes in AMPA receptor trafficking and dendritic growth (Seeburg et al., 2008; Lee et al., 2011).

A second pathway which has received significant attention as a possible link between sleep and homeostatic downscaling is the Homer1a pathway. *Homer1a* is an immediate early gene and the short isoform of constitutively active Homer proteins. The constitutive Homer proteins act as scaffolds which bring together a complex including NMDA receptors and mGluR5 receptors at the post-synaptic density. In response to increased neuronal activity, the shorter Homer1a protein acts as a dominant negative isoform, which can disrupt this complex (Kammermeier and Worley, 2007). Loss of Homer1a disrupts homeostatic downscaling (Siddoway et al., 2014), and restoring its expression leads to decreased AMPA and metabotropic glutamate receptor expression at the post-synaptic density (Hu et al., 2010). Recent gene expression studies have shown that *homer1a* expression increases across the brain in response to SD (Nelson et al., 2004; Mackiewicz et al., 2007) and the genetic locus for *homer1a* has been implicated in the homeostatic regulation of NREM slow wave activity (Mackiewicz et al., 2008). Diering et al. (2017) recently found that Homer1a protein abundance at synapses rapidly increases during SD. If we assume that Homer1a localization at the synapse results in downscaling, this finding would suggest that downscaling occurs during wake. However, the authors also reported that reductions in synaptic GluA1 and GluA2 during the sleep phase of the circadian cycle were dependent on Homer1a. To reconcile these findings, the authors hypothesized that Homer1a mobilization to the synapse is gated by both norepinephrine and adenosine levels. They speculated that during wake, high levels of norepinephrine maintain Homer1a outside the synapse; reduced norepinephrine and increased adenosine levels lead to delivery of Homer1a to the post-synaptic density during sleep. In support of this idea, treating mice with the norepinephrine reuptake inhibitor d-amphetamine (or an A1 adenosine receptor antagonist) reduced synaptic Homer1a levels, while treating them with norepinephrine receptor antagonists increased synaptic Homer1a (Diering et al., 2017). The authors of the study argued that this represented a plausible mechanism whereby prolonged wakefulness could lead to subsequent synaptic downscaling during sleep. However, it is worth noting that in this study, the observed sleep-associated reduction in GluA1 levels preferentially occurred among spines with the highest baseline levels (i.e., it was not global). Indeed, some spines showed increases in GluA1 levels. Taken together with other evidence showing that synaptic weakening is heterogeneous during sleep (de Vivo et al., 2017), these data actually argue *against* true synaptic downscaling as a mechanism for sleep-dependent synaptic changes.

Intriguingly, sleep and wakefulness may have differential effects on so-called “upscaling”—which globally increases synaptic strengths in response to decreased network activity. Hengen et al. evoked homeostatic plasticity in freely behaving mice via monocular lid suture, leading to reduced visual cortex activity. The authors found that homeostatic increases in spontaneous firing rate after this treatment were primarily expressed across bouts of wake, with longer wake epochs resulting in greater firing rate increases (Hengen et al., 2016). The authors concluded that cellular mechanisms responsible for upscaling are active during wake, and inhibited by sleep.

#### Homeostatic maintenance of excitatory/inhibitory (E/I) balance

Numerous studies have indicated that homeostatic responses to increased network activity may also involve modifications to GABAergic synapses, effecting a change in the balance of network excitation and inhibition. Following periods of overactivity, inhibitory synapses on pyramidal neurons have been shown to undergo presynaptic and post-synaptic enhancements, including increases in presynaptic GAD65 and GABAA receptor surface expression (Peng et al., 2010; Rannals and Kapur, 2011). Recent data suggest that GABAA receptor surface expression is increased on cortical pyramidal neurons *in vivo* in response to brief SD (Del Cid-Pellitero et al., 2017). Homeostatic increases in GABAA receptor expression have recently been linked to changes in the localization of gephyrin, a scaffolding protein that anchors GABAA receptors to the inhibitory PSD (Flores et al., 2015). Flores et al. found that the number and size of gephyrin clusters increase in pyramidal neurons following prolonged network activity. These clusters colocalize with GAD67 and are accompanied by increases in miniature inhibitory post-synaptic current (mIPSC) amplitude and frequency in response to prolonged depolarization of pyramidal neurons. Recent data suggest that this mechanism may be directly affected by sleep vs. wake. For example, *gephyrin* mRNA levels are higher in the brain after a period of sleep relative to a period of wake (Cirelli et al., 2004). Gephyrin is stabilized at the synapse by phosphorylation by CDK5 (Kalbouneh et al., 2014), which as mentioned above may be activated preferentially during sleep (Diering et al., 2017).

Glutamatergic synapses on inhibitory interneurons may also be potentiated in response to increased network activity, leading to increased feedback inhibition within the network. Chang et al. found that network overactivity results in significantly increased expression of the immediate early gene *narp* and NARP protein in pyramidal neurons. The authors found that subsequently, NARP is released presynaptically in parvalbumin-expressing interneurons, causing increases in surface expression of GluA4 containing AMPA receptors (Chang et al., 2010). *Narp* expression is increased throughout the brain after a period of wakefulness (Cirelli et al., 2004). Given the differential regulation of *narp* and *gephyrin* expression by wakefulness/sleep, it is possible they maintain network stability by modulating inhibitory activity at different time points to alter E/I balance. Whether these pathways are evoked *in vivo* as a consequence of learning-associated synaptic potentiation is unknown. However, sleep-associated changes in the number of inhibitory synapses have been observed in the cortex, as described above (Del Cid-Pellitero et al., 2017). Taken together, there are numerous alternate pathways by which sleep could regulate homeostatic changes in neural circuits in response to augmented network activity.

#### NREM oscillation-driven synaptic weakening

Proponents of SHY have speculated that synaptic weakening is mechanistically linked to the synchronous, low-frequency rhythms (slow wave activity; SWA) that synchronize thalamocortical and hippocampo-cortical activity patterns during NREM sleep (Buzsaki et al., 2003; Sirota et al., 2003). They argue that, like synaptic strength, SWA is homeostatically regulated. With increased time spent awake (and according to the hypothesis, more opportunity for synaptic potentiation), SWA during subsequent NREM sleep is significantly enhanced. After an initial period of recovery sleep, this enhanced SWA returns to baseline—a process which is speculated to reflect a renormalization of synaptic strength to levels seen before waking experience. Thus, according to SHY proponents, SWA homeostasis and synaptic homeostasis go hand in hand. Beyond this, numerous studies have also indicated that NREM SWA is selectively enhanced in cortical areas that are preferentially activated (e.g., by learning) during prior wake periods. Conversely, SWA is selectively decreased in cortical areas that are less active during prior waking experience (Huber et al., 2004, 2006). In the context of SHY, this has been interpreted as evidence for a causal role of SWA thalamocortical activity patterns in promoting synaptic weakening.

There is evidence that experimentally-generated firing patterns (analogous to those occurring during SWA) can cause LTD of glutamatergic synapses *in vitro*. A variety of paradigms have been used to emulate the activity patterns seen in thalamocortical and hippocampal circuits during NREM. One of these is low frequency stimulation—trains of single spikes or short bursts, occurring at frequencies between 1 and 3 Hz. This rhythmic pattern of activity mimics that generally seen in both hippocampal and cortical circuits during NREM SWA *in vivo*. However, numerous labs have reported that low frequency stimulation (i.e., 1 Hz trains or burst stimuli, which can induce LTD of *in vitro*) is insufficient for *in vivo* LTD induction in either the hippocampus (Errington et al., 1995; Abraham et al., 1999) or cortex (Jiang et al., 2003; Hager and Dringenberg, 2010). In contrast, higher-frequency stimulation can reliably induce LTP in hippocampal and thalamocortical circuits *in vivo* (Heynen and Bear, 2001; Whitlock et al., 2006; Cooke and Bear, 2010).

It is unclear why many stimulation protocols induce LTD less robustly *in vivo*, while LTP is more easily induced. In neural circuits where it has been studied, the level of spontaneous activity (which varies with brain state) seems to be a critical variable for both LTD induction and maintenance. For example, LTD can be induced more reliably in the cortex *in vivo* if animals are deeply anesthetized (Hager and Dringenberg, 2010). This effect of anesthesia can be blocked by stimulation of the pedunculopontine (PPT) nucleus (which is wake-active, and provides cholinergic input to the thalamus) (Stewart and Dringenberg, 2016). Because PPT activity is generally low during NREM relative to wake (Jones, 2005; Mena-Segovia et al., 2008), and because spontaneous thalamocortical activity is generally lower in NREM than in REM or wake (Vyazovskiy et al., 2009), it is tempting to speculate that NREM sleep provides ideal (and necessary) state conditions for *in vivo* LTD induction. NREM thalamocortical activation patterns also provide another feature that might be ideally suited for inducing LTD—burst mode firing. Bursts of presynaptic action potentials paired with post-synaptic EPSPs reliably induce LTD of cortical glutamateric synapses *in vitro* (Birtoli and Ulrich, 2004; Czarnecki et al., 2007). Bursts of action potentials with no post-synaptic EPSPs may also reduce subsequent glutamatergic neurotransmission by driving elimination of post-synaptic calcium-permeable AMPA receptors (Lante et al., 2011). EPSP-paired bursting can elicit LTD at any time of day (after periods of more sleep or more wake), while unpaired bursting can elicit synaptic depression throughout the day. This suggests that at least two forms of activity-dependent LTD may be expressed at cortical synapses, and these are differentially affected by sleep history. Since these studies were carried out *ex vivo*, and in cortical slices taken from juvenile animals, future studies will have to address how these mechanisms are affected *in vivo* and into adulthood (when rates of synaptic pruning are generally reduced).

There is also evidence that over the long term (24 h, vs. minutes for inducing LTD), low-frequency stimulation may also activate the same cellular pathways involved in homeostatic synaptic downscaling. (Goold and Nicoll, 2010) recently demonstrated that prolonged optogenetic low-frequency stimulation of individual hippocampal neurons led to both cell-autonomous downscaling of NMDA and AMPA receptor-mediated currents, and dramatic synaptic pruning. These effects were mediated post-synaptically (i.e., in optogenetically-stimulated neurons) via CaMKK and CaMKIV, and removal of GluA2-containing AMPA receptors and NMDA receptors (Goold and Nicoll, 2010).

Despite these data, it is worth noting that NREM sleep is characterized by other network activity features in addition to SWA. In thalamocortical circuits, sleep spindles emerge as 7–15 Hz coherent network oscillations, which are expressed as discrete waxing-and-waning events during NREM (Clawson et al., 2016). Recent *ex vivo* studies have aimed at mimicking patterns of activity during NREM spindles to determine effects on synaptic strength. Rosanova and Ulrich recorded activity from neurons in somatosensory cortex during spindles, and used this pattern to drive presynaptic activity in layer 2/3 while recording post-synaptic responses in layer 5 (Rosanova and Ulrich, 2005). The authors found that when this pattern was repeated at a frequency similar to the frequency of NREM spindle occurrence, post-synaptic responses were potentiated. Moreover, a synthetic spindle activity pattern (presynaptic bursts delivered at 10 Hz) likewise drove post-synaptic LTP. Thus, NREM network oscillations of different frequencies may have divergent effects on synaptic strength in cortical circuits.

#### REM-associated reductions in neural network activity

Proponents of SHY have emphasized the potential mechanistic link between NREM SWA and synaptic weakening. However, most measurements of molecular, functional and structural synaptic changes have been measured after periods of sleep, which includes REM. REM sleep constitutes roughly 10–30% of total sleep time in adult mammals, depending on species. Intriguingly, the proportion of time spent in REM sleep across species has been linked to brain mass (Lesku et al., 2006, 2008). Studies evaluating sleep time across phylogeny have not found a similar link between NREM sleep time and brain size. This begs the question—could REM, rather than NREM SWA, mediate synaptic weakening across intervals of sleep? There are some experimental data that would suggest that this is possible. Firing rates in both cortical (Durkin and Aton, 2016; Watson et al., 2016) and hippocampal neurons (Grosmark et al., 2012; Miyawaki and Diba, 2016) decrease consistently across bouts of REM. Firing decreases are proportional to REM bout duration in the cortex (Watson et al., 2016) and to the amplitude of locally-generated theta (4–12 Hz) oscillations in the hippocampus (Grosmark et al., 2012; Miyawaki and Diba, 2016). A recent fMRI study (van der Helm et al., 2011) indicated that overnight decreases in amygdala functional responses to an emotionally arousing task are related to REM-associated EEG activity. More recently, a study measuring overall levels of cortical neural activity (with wide-field imaging of calcium signals) found that activity is globally reduced in the cortex (in all cortical layers) across bouts of REM (Niethard et al., 2016). In support of the idea that these functional changes are related to synaptic weakening, a recent *in vivo* imaging study demonstrated that the selective elimination of newly-formed dendritic spines is blocked by REM-targeted SD (but not NREM disruption) (Li et al., 2017). What features of REM could mediate synaptic weakening? Recent calcium imaging data indicates that the relative activity of fast spiking interneurons to pyramidal neurons is significantly higher during REM relative to NREM and wake (Niethard et al., 2016). Thus, REM may alter the E/I balance of neural networks, which could bias plasticity at glutamatergic synapses, to favor synaptic weakening. Alternatively, the relative high levels of cortical and hippocampal acetylcholine release (and simultaneous relative low levels of norepinephrine, serotonin and dopamine release) during REM may bias circuit plasticity in favor of spike timing-based LTD (see above).

#### Glial regulation of synaptic function

Multiple lines of evidence have indicated that the biological support system surrounding neurons is significantly affected by sleep and wake states. Recent studies focused on the so-called “glymphatic” system have shown that interstitial space in the cortex increases significantly during NREM sleep, over a timescale of minutes (Xie et al., 2013). This process, mediated by astrocytic regulation of peri-arterial flow rates, is thought to promote both delivery of nutrients, and clearance of potentially harmful metabolic waste from the brain. Such a mechanism could affect synaptic function in myriad ways. For example, levels of extracellular glucose decline across bouts of wake and REM, and increase at the transition from wake to NREM sleep (Dash et al., 2013). At the same time, lactate accumulates in the brain (as a product of glycolysis) during wake (and also during REM sleep) (Naylor et al., 2012) and is cleared by the glymphatic system during NREM sleep (Lundgaard et al., 2017). Because at high enough concentrations lactate can potentiate NMDA receptor-mediated currents, leading to downstream changes in the expression of plasticity-related genes in the brain (Yang, J. et al., 2014), this mechanism could potentially mediate sleep-dependent synaptic weakening.

Sleep changes not only the volume of the brain's interstitial space, but also its ionic content. A more recent study demonstrated that the extracellular concentrations of calcium, magnesium, and protons increases (and the concentration of potassium decreases) in the cortex as animals transition from wake to NREM sleep (Ding et al., 2016). As might be expected, such changes directly impact the mode of firing in cortical neurons (and ECoG activity), but it remains unclear whether they also directly impact synaptic function and synaptic strength. Available data suggests that changes in the concentrations of these ions (like those that accompany wake-to-sleep transitions) can lead either to a selective increase in excitatory transmission (resulting in increased E/I balance) (Meeks and Mennerick, 2004) or to synaptic potentiation (Harsanyi and Friedlander, 1997; Hess, 2002; Abumaria et al., 2011; Du et al., 2014). Intriguingly, these extracellular ion concentration changes could all be mediated by astrocytes (Verkhratsky and Nedergaard, 2014) and could in turn impact the activity pattern of surrounding neurons (Ding et al., 2016). Indeed, recent experimental data has shown that optogenetic hyperpolarization of astrocytes leads to changes in local field potential (LFP) activity similar to that seen as animals transition to NREM sleep (Poskanzer and Yuste, 2016).

While the precise cellular mechanisms underlying all of these effects are generally unknown, it is clear from studies using cell type-specific mRNA profiling (i.e., translating ribosome affinity purification; TRAP) that sleep and wake affect a variety of cellular processes in both astrocytes (Bellesi et al., 2015) and oligodendrocytes (Bellesi et al., 2013). One speculative mechanism is based on the fact that ATP released from neurons during heightened network activity activates purinergic receptors on microglia, leading to release of interleukin 1 (IL1) and tumor necrosis factor-alpha (TNFα) (Hide et al., 2000; Shieh et al., 2014). Because IL1 and TNFα can induce NREM sleep, this signaling mechanism has been hypothesized to mediate both sleep homeostasis after extended wake, and local, use-dependent changes in NREM thalamocortical oscillations (Kreuger et al., 2011). Some have speculated that this same signaling pathway may also mediate sleep-associated synaptic weakening. However, because the *in vitro* effects of TNFα on glutamatergic (Beattie et al., 2002; Karrer et al., 2015) and GABAergic (Stellwagen et al., 2005; Pribiag and Stellwagen, 2013) synapses are diverse, it remains unclear whether glial-derived TNFα signaling offers a plausible molecular mechanism for synaptic weakening during sleep.

## Part 2: a counterpoint to SHY—a role for synaptic strengthening in the cognitive benefits of sleep

**Figure 2:**
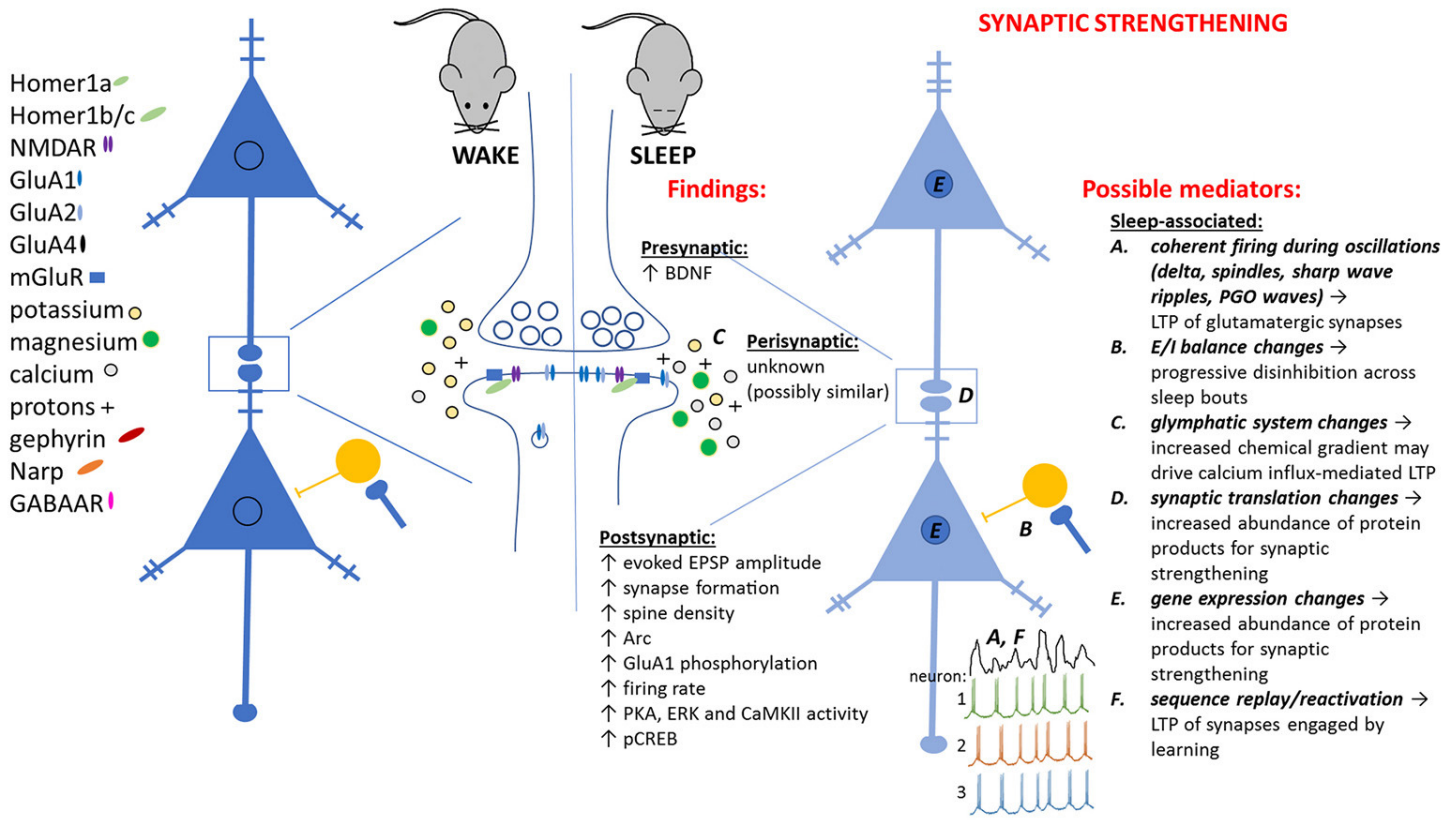
Observed pre- and post-synaptic changes attributed to sleep-dependent synaptic strengthening, and potential sleep-dependent mechanisms.

An increasing body of data has presented counterpoints to SHY (see Table 1). These studies have primarily been aimed at investigating the neurobiological correlates of sleep-dependent learning and memory storage, following novel learning experiences during wake. Surprisingly, many of these investigations have found evidence of synaptic strengthening, not weakening, across periods of post-learning sleep (see Table 1 and Figure 2). Thus, one possibility, which we put forth here, is that different types of synaptic plasticity (not synaptic weakening alone) may be promoted during sleep, depending on the circumstances of an animal's prior waking experience. Here we will briefly describe what is known about some example cases in which synaptic strengthening occurs during sleep, leading to adaptive changes in brain function.

### Contextual fear memory (CFM)

CFM is a well-studied form of long-lasting memory, which can last days or even months in mice following a single learning experience. As such, it has been described as analogous to episodic memory in humans. CFM consolidation is disrupted by SD in the hours following single-trial contextual fear conditioning (CFC) (Graves et al., 2003; Prince et al., 2014). CFM consolidation relies on neural activity in hippocampal area CA1 during the same window of time post-CFC (Daumas et al., 2005); a reasonable conclusion is that network activity patterns in CA1 during sleep play an essential role in memory storage. Recent work from our lab (Ognjanovski et al., 2014) has demonstrated that during this window of time, CA1 neuronal firing and the amplitude of network oscillations are enhanced; these changes from baseline are present during both NREM and REM sleep. Furthermore, functional connectivity relationships between CA1 neurons (quantified based on relative spike timing among stably-recorded neurons) are selectively enhanced during NREM sleep following learning. This change is present across NREM over the entire 24 h between CFC and fear memory testing—suggesting a plausible neural substrate for memory storage. More recently, we found an experimental strategy to disrupt the post-CFC enhancement in NREM and REM CA1 oscillations—by selective inhibition of parvalbumin-expressing (PV+) interneurons in the hours following CFC. We found that pharmacogenetic inhibition of PV+ interneurons disrupts both stabilization of functional connectivity patterns in CA1 during NREM, and CFM consolidation (Ognjanovski et al., 2017). By optogenetically activating PV+ interneurons in a rhythmic fashion (mimicking rhythms enhanced during post-CFC sleep), we were able to both stabilize and strengthen functional connectivity relationships between neurons across CA1. Taken together, this suggests that sleep oscillations which are augmented in the hippocampus after learning promote long-term memory formation via synaptic strengthening, rather than synaptic weakening. CFM consolidation is linked mechanistically to LTP of glutamatergic synapses in CA1, for several reasons. First, behavioral manipulations such as SD that interfere with CFM consolidation also disrupt Schaeffer collateral LTP in CA1 (Vecsey et al., 2009). Second, disruption of intracellular pathways required for LTP in CA1 also disrupt CFM consolidation (Abel et al., 1997; Atkins et al., 1998; Vecsey et al., 2009; Havekes et al., 2016). Third, intracellular pathways required for LTP are activated in the hippocampus immediately following CFM (Atkins et al., 1998). Finally, experimental manipulations that enhance hippocampal LTP also enhance CFM consolidation (Abrari et al., 2009). Thus, all available evidence suggests that in the case of CFM consolidation, sleep activates cellular pathways in the hippocampus to induce synaptic potentiation (not downscaling), in order to promote memory formation. Taken together, these data present a clear non-SHY synaptic mechanism underlying specific cognitive benefits of sleep.

### Motor cortex plasticity after learning

A large number of studies using human subjects have shown benefits of sleep for sensorimotor performance following learning a new sensorimotor task (Doyon, 2008; King et al., 2017). Depending on the specific motor task involved, these studies have linked the benefits of sleep on motor performance to changes in local slow wave and spindle oscillations in supplementary motor cortical areas following learning (Tamaki et al., 2013), changes in SWA in parietal cortical areas involved in multisensory spatial information processing (Huber et al., 2004), and total post-learning NREM sleep time (Robertson et al., 2004). Additionally, experimental disruption of cortical SWA following learning has been shown to disrupt consolidation of at least some forms of sensorimotor learning (Landsness et al., 2009).

Recent studies using repeated functional brain imaging during motor task acquisition demonstrated that a correlate of sleep-dependent performance enhancement is an increase in task-related brain activity in corticostriatal and cerebellar motor systems following a period of sleep (Debas et al., 2010; Fogel et al., 2017). This increase in task representation in the brain after a period of post-learning sleep is suggestive of synaptic strengthening, insofar as BOLD signal changes reflect changes in the extent of synaptic activity. A more definitive demonstration of sleep-dependent synaptic strengthening (or at least synaptic growth) during NREM sleep occurs following motor learning in mouse primary motor cortex (M1) (Yang, G. et al., 2014). In their recent study, Yang and colleagues demonstrated that SD (but not REM-selective SD) disrupted formation of new dendritic spines in M1 layer 5 (i.e., output) pyramidal neurons in the hours after a period of motor learning.

### Ocular dominance plasticity (ODP) and orientation-specific response potentiation (OSRP) in the visual cortex

There are multiple examples of synaptic strengthening during sleep from the visual system following novel visual experiences. One is the effect that sleep has in the primary visual cortex (V1) in the context of ocular dominance plasticity (ODP)—a well-studied form of cortical response plasticity initiated by loss of visual input to the cortex from one of the two eyes. ODP is an adaptive response that shifts V1 neurons' visual responsiveness from binocularity to favoring the spared eye. The role of sleep in promoting this process has been studied for nearly two decades. In 2001, Frank et al. initially reported that during a sensitive period of post-natal development, a modest shift in visual responses occurs in cat V1 following a brief (6-h) period of monocular visual experience (Frank et al., 2001). This shift is effectively reversed by a subsequent 6-h period of SD (without further visual input), but is significantly augmented by 6 h of subsequent *ad lib* sleep. The mechanism mediating this sleep-dependent enhancement of ODP involves activation of LTP-mediating kinase pathways, relies on NMDA receptor activation and protein synthesis, and causes an enhancement of V1 neurons' firing rate responses to spared-eye stimulation (Aton et al., 2009a; Seibt et al., 2012; Dumoulin et al., 2015). These changes are associated with changes in V1 network activity during sleep in the hours following monocular experience—including reduced fast-spiking interneuron firing (which occurs specifically in cortical areas representing the spared eye), increased principal neuron firing, and increased neuronal firing coherence with both slow wave and spindle oscillations in NREM sleep (Aton et al., 2009a, 2013).

While ODP (1) is induced by a loss of visual input, and (2) is most robustly induced during a relatively brief post-natal window, orientation-specific response potentiation (OSRP) is expressed in adulthood in mouse V1 in response to specific patterns of visual input (Frenkel et al., 2006). Our laboratory has shown that following a brief period of exposure to an oriented grating stimulus (lasting 30–60 min), OSRP is expressed in V1 neurons as an enhanced response to stimuli of the same orientation. This response change is not present immediately following the visual experience, but is only seen after 6–12 h of subsequent sleep (Aton et al., 2014; Durkin and Aton, 2016). OSRP is blocked by post-stimulus SD, and is proportional to post-stimulus NREM and REM sleep time (Aton et al., 2014; Durkin and Aton, 2016). As is true for both CFM consolidation and ODP in V1, OSRP consolidation is associated with an increase in firing rate among V1 neurons in the hours following experience (which apparently occurs across bouts of NREM and REM, not wake) (Durkin and Aton, 2016), and is proportional to an increase in phase-locking of V1 neuronal firing to NREM oscillations (Aton et al., 2014). The expression of OSRP is linked to clear, stimulus-selective enhancement in firing rate responses to visual stimulation in V1 neurons, suggestive of synaptic potentiation (Durkin and Aton, 2016). This interpretation is consistent with studies of the underlying mechanisms of OSRP. For example, OSRP is blocked by interference with cellular pathways required for LTP of glutamatergic synapses (Frenkel et al., 2006). Further, *in vivo* thalamocortical LTP induction (with high-frequency LGN stimulation) occludes subsequent induction of OSRP, and OSRP induction occludes subsequent LTP between LGN and V1 (Cooke and Bear, 2010). Together, these data suggest a common mechanism between LTP of thalamic relay synapses in the cortex and sleep-dependent OSRP consolidation.

### A data-driven alternative to SHY

What do all of these exceptions to SHY have in common? In all cases, the animal is being trained on a novel task, or having a novel experience, immediately prior to sleep. Based on available data, we propose an alternative to SHY—an alternative that applies to situations where sleep follows a learning experience in wake. In this scenario, we propose that circuit-specific changes in gene expression and protein translation during wake lead to subsequent changes in network activity during subsequent sleep. These changes in network activity support strengthening of at least a subset of network glutamatergic synapses (see Figure 2). We speculate that, consistent with the examples described above, sleep-dependent synaptic strengthening is essential for the cognitive benefits of sleep. In contrast (and in contradiction to SHY) sleep-associated synaptic weakening may not play a critical role in promoting cognitive function. Thus far, there is scant evidence to suggest that sleep-dependent learning and memory processes are related to synaptic weakening, and none to suggest they are associated with homeostatic downscaling.

The forms of sleep-dependent plasticity described above have several features in common. They are all associated with circuit-specific changes in network activity including: (1) increases in neuronal firing rate, (2) amplified NREM (and occasionally, REM) oscillations, and (3) phase-locking of neuronal firing to these oscillations. Current data suggest that these changes are the direct result of learning experience during prior wakefulness. We speculate that while synaptic weakening may occur across sleep in the absence of learning (e.g., for mice housed in standard conditions), post-learning changes to network activity in the sleeping brain can support synaptic strengthening.

### Synaptic strengthening in NREM sleep

SHY proponents have linked synaptic weakening during sleep to NREM oscillations. In the cases described above, however, NREM oscillations (and neuronal firing coherence with them) have been linked to synaptic strengthening and growth, resulting in either memory consolidation, adaptive sensory plasticity, or motor learning. Might NREM oscillations differentially affect synaptic strength (bringing it either up or down within a given circuit) depending on prior experience? This is a possibility. Indeed, work from our own lab suggests that this may be the case. One example of this is the firing rate changes that occur in individual V1 neurons after a period in of dark exposure (i.e., no visual experience) vs. patterned visual experience. In the former case, an increase is seen across bouts of wake, no change across NREM bouts, and a decrease across REM bouts; in the latter, firing rates increase selectively during NREM and REM sleep bouts (but not wake). Another example comes from the rat somatosensory cortex, where prior experience with a spatio-tactile task (novel object exploration) led to selective increases in firing rate during the next 3 h of subsequent sleep (Ribeiro et al., 2007).

The idea that NREM oscillations play a critical role in patterning brain plasticity was recently reinforced by findings from a study using optogenetics to mimic NREM slow wave oscillations (with simultaneous 2 Hz stimulation of mouse somatosensory and motor cortex) following training on a somatosensory perceptual task (Miyamoto et al., 2016). The authors found that synchronous stimulation of the two areas rescued perceptual learning in mice from deficits induced by post-learning SD. Chauvette et al. (2012) recently attempted to clarify the immediate effects of NREM slow wave oscillations on synaptic strength in the cat cortex, *in vivo* and *in vitro*. The authors found that cortical evoked potentials were enhanced selectively across periods of NREM sleep (but not across periods of wake or REM). They also found that presynaptic stimulation patterned to mimic that seen in SWA (but not stimulation patterned to mimic wake activity) led to long-term increases in EPSP amplitude in cortical neurons. A more recent study (Sadowski et al., 2016) showed that in the hippocampus, neuronal firing in the context of a sharp wave ripple oscillation can directly promote LTP *in vitro*. A reasonable conclusion is that the firing patterns evoked by NREM oscillations are conducive to synaptic potentiation.

### Synaptic strengthening in REM sleep

The majority of recent work focused on sleep-dependent plasticity has emphasized a role for NREM sleep in the process. However, it is worth noting that a number of findings have suggested that synaptic strengthening can occur specifically in REM sleep. For example, either brief (i.e., hours-long) or long term (days-long) periods of REM-targeted SD, can disrupt subsequent induction and maintenance of hippocampal CA1 LTP (Ishikawa et al., 2006; Ravassard et al., 2016). Related to this deficit, brief REM-targeted SD in the hours following learning is sufficient to disrupt some forms of hippocampally-mediated memory consolidation (Datta and O'Malley, 2013; Ravassard et al., 2016). These effects are related to changes in PKA and CREB signaling, and changes in the expression of Arc and BDNF, in the hippocampus and in other areas involved in mnemonic processing (Ribeiro et al., 1999; Ulloor and Datta, 2005). While the systems- and network-level mechanisms responsible for REM's influence on hippocampal LTP and hippocampally-mediated memory formation are still largely unknown, memory consolidation in some REM-dependent tasks is correlated with the occurrence of pontine-geniculate-occipital (PGO) waves (which occur preferentially at the transition from NREM to REM and during REM). Activation of pontine circuitry that promotes PGO waves (leading to increased PGO wave occurrence) can rescue certain forms of REM-dependent memory in the context of REM SD (Mavanji and Datta, 2003). More recently, REM sleep was also shown to play a critical role in the consolidation of ODP in cat V1. The shift in visual responses in favor of the spared eye was greatly reduced when REM sleep was selectively deprived in the hours following monocular visual experience (Bridi et al., 2015). REM SD also disrupted visual experience-induced enhancements in LTP-mediating kinase (i.e., ERK) activity in V1 during post-experience sleep. Furthermore, neuronal firing rates are increased during post-learning REM, in both mouse hippocampus in the hours after single-trial CFC (Ognjanovski et al., 2014, 2017), and in mouse V1 following induction of OSRP (Aton et al., 2014; Durkin and Aton, 2016). Indeed, changes in firing rate in V1 neurons increase more across bouts of REM than across bouts of NREM in the hours following novel visual experience (Durkin and Aton, 2016). These changes, like changes in the occurrence of PGO waves, and the expression of many immediate-early genes involved in synaptic potentiation, are dependent on experience during prior wake (Ribeiro et al., 1999; Datta, 2000; Aton et al., 2014; Ognjanovski et al., 2014; Durkin and Aton, 2016).

## Part 3: the function of sleep-dependent “replay” of network activity patterns

### What Is replay?

A great deal of recent data suggest that reactivation of task-associated neuronal ensemble activity patterns occurs during subsequent sleep, leading to speculation that this reactivation drives sleep-dependent memory consolidation. One barrier to our understanding of the function of reactivation in neural circuits is that it has been defined using a variety of conceptual and quantitative means. Early studies by Pavlides and Winson (1989) defined task-associated activity as temporally-correlated firing among neuron pairs during experience. Using this definition, Pavlides and Winston (and others) first described sleep-dependent reactivation of place cells following exploration of new environments (Pavlides and Winson, 1989; Wilson and McNaughton, 1994). Other recent studies have described stabilization of functional communication patterns (based on spike timing between neurons) during NREM sleep following single-trial learning (Ognjanovski et al., 2014, 2017) or selective reactivation during REM sleep of neurons activated by novel sensory experience (Bridi et al., 2015). Because such network-level changes occur during sleep following a single learning event, they are plausible substrates for promoting synaptic plasticity.

In recent years, however, the term “replay” has been used in reference to precise sequential reactivation of neurons engaged sequentially during a spatial task. For technical reasons, the majority of these studies have focused on the reactivation of hippocampal place cells—neurons with spatially selective receptive fields. As an animal traverses an environment, place cell neurons fire to encode its changing location, creating sequential patterns of activation that reflect its trajectory. Using this sequence as a template, one can quantify replay events during subsequent REM or NREM sleep (Louie and Wilson, 2001; Ji and Wilson, 2007). An essential component of experiments measuring sleep-associated sequential replay is the generation of a reliable, repeatable behavioral sequence. In studies using rodents, this usually requires weeks of repetitive training on a spatial task. Because the animals in these studies are carrying out a familiar (rather than new) task prior to measurements of replay events, the relationship between sequence reactivation and new memory formation is not generally clear.

### What causes replay?

How does sequential replay occur? One parsimonious interpretation of data involving highly trained animals engaged in a repetitive spatial task is that the sequence of neuronal activation is simply “hard-wired” due to the strength of connections between neurons in the ensemble. This might explain the fact that replay, relative to sequential activation during behavior, tends to be time-compressed. If neurons in the ensemble were synaptically connected and played a strong causal role in driving one another's firing, they would fire sequentially during spontaneous activity with minimal synaptic delays. It would also explain the fact that replay events can occur in practically any brain state (with reports of replay in NREM, REM, and wake) (Sadowski et al., 2011). Finally, if the neurons were reciprocally connected, this interpretation could also explain the occurrence of reverse replay events (where the sequence of neuronal activation is opposite that seen during behavior) (Diba and Buzsaki, 2007). A related mechanism that has been proposed (the so-called “lingering excitability model”) (Atherton et al., 2015) is based on the relative excitability of place cells, where neurons that have been most recently activated (i.e., by the animal's recent presence in their respective place fields) are more likely to initiate a sequential (forward or reverse) replay event. This would explain the apparent hysteresis of replay events. For example, sequences of activity that have occurred more frequently in an animal's recent past (during behavior) are more likely to replay when the behavior ends (Atherton et al., 2015). Furthermore, during pauses in a run, replay sequences are most likely to initiate with the firing of the place cell representing the space that the animal currently occupies (Atherton et al., 2015). However, neither of these explains another phenomenon related to replay—the occurrence of sequential activity patterns *before* a set of place cells is sequentially activated during behavior (so-called “preplay”). Preplay maps of place field activation have been reported to predict future trajectories, *despite occurring prior to actual experience*. Recently, a study by the Foster lab questioned the occurrence of preplay events, suggesting that they may result from a statistical anomaly. Using a larger sample of neurons, and slightly different quantitative methods, the authors were unable to find evidence of preplay events (Silva et al., 2015). Nonetheless, reports of reverse replay and preplay, which can at times represent never before experienced behavioral sequences, brings into question the hypothesis that replay promotes memory consolidation.

### Does replay play a role in memory consolidation?

Despite the caveats outlined above, various arguments have been put forward in support of the idea that sequential replay could promote memory consolidation, particularly in the context of post-learning sleep. During replay events, sequential patterns of neuronal activation are compressed to a time scale compatible with STDP. Such compressed replay occurrences preferentially occur during sharp wave ripple events, which (1) occur preferentially in the hippocampus during NREM sleep and (2) have themselves been linked to memory formation (Girardeau et al., 2009). Thus, it has been argued that replay offers an instructive mechanism for promoting formation of specific memories, by altering the strength of connections between neurons sequentially engaged during waking experience. Coordinated replay between brain areas (typically hippocampus and cortex) during sleep is proposed to be a critical mediator of systems memory consolidation (Aton et al., 2009b; Aton, 2013). Sequential replay of neuronal activity patterns has been seen in cortical structures like the prefrontal cortex following spatial task performance (Euston et al., 2007), and coordinated hippocampal and cortical sequential replay has been described in the context of spatial learning (i.e., maze running) (Ji and Wilson, 2007). However, there is currently no evidence that such sequence reactivation is temporally associated with, or critical for, *de novo* memory formation. In contrast, there are suggestions that sleep-dependent, coordinated reactivation of specific neuronal populations in hippocampus and cortex may promote information transfer between the two structures. For example, a recent study using dual-site recording found that NREM sharp wave ripple events in hippocampus triggered reactivation of neuronal ensembles in prefrontal cortex that were co-activated during prior spatial task learning (Peyrache et al., 2009). Intriguingly, while early data suggested preferential information flow from hippocampus to cortex during NREM sharp wave ripple events (Buzsaki, 1996; Wierzynski et al., 2009), more recent findings suggest that activity patterns in the cortex can inform the activation pattern in the hippocampus during these events (Rothschild et al., 2017). Because during NREM, hippocampal sharp wave ripples are coordinated with neocortical slow waves (Sirota et al., 2003; Molle et al., 2006), this suggests that during NREM-associated oscillations there is a true dialogue between neurons in hippocampal and thalamocortical circuits. Such a dialogue may promote the formation of widely distributed memory traces in the context of consolidation.

A major unresolved question for the field is whether replay or reactivation promotes synaptic plasticity and long-term memory formation. Because the memories in question are associated with activity in sparsely-distributed neuronal populations, direct measurement of functional connectivity (i.e., mEPSC amplitude or frequency) or anatomical plasticity (i.e., spine size or density) associated with memory consolidation is a technical challenge. A few studies have attempted to resolve whether replay events can be disrupted by NMDA receptor antagonism *in vivo* (Dupret et al., 2010; Silva et al., 2015). These studies are illuminating for multiple reasons. First, such antagonism is almost universally amnestic for the types of (episodic or spatial) memories typically under study with respect to replay. Second, since many forms of Hebbian plasticity rely on NMDA receptor signaling, this treatment should disrupt any events relying on, for example, LTP. Data from these studies suggests that replay/reactivation events related to newly-learned trajectories or locations is lost in the absence of NMDA receptor signaling (Dupret et al., 2010; Silva et al., 2015). This suggests that replay occurrence is at least related to new memory formation.

### Summary and future directions

While a variety of lines of evidence point to a role for sleep in promoting widespread synaptic weakening, two major questions remain unresolved. First, it is unclear whether brain activity patterns associated with either REM or NREM sleep are essential for promoting synaptic weakening, and if so, how this is accomplished. Second and more importantly, it is unclear whether any of the cognitive benefits of sleep are related to this process. Available data from studies addressing the effects of sleep on synaptic function in the context of sleep-dependent episodic or procedural memory formation suggest that strengthening, rather than weakening, could play a key role. However, even in these cases (where functional and behavioral effects of sleep can be directly measured) it remains unclear what aspects of the sleeping brain state are critical for promoting plasticity. Future studies should take advantage of what is known about basic cellular plasticity mechanisms (only some of which are described here) to assess sleep-dependent mechanisms. Recent technical advances in optogenetics, long-term brain imaging, and long-term electrophysiological recording may help link the activity patterns associated with sleep to these specific cellular pathways, to clarify why and how sleep benefits cognition.

## Author contributions

SA and CP determined the content and wrote the manuscript.

### Conflict of interest statement

The authors declare that the research was conducted in the absence of any commercial or financial relationships that could be construed as a potential conflict of interest.
